# Supplementary material for: Trauma Recovery Rubric: A Mixed-Method Analysis of Trauma Recovery Pathways in Four Countries
Source: Int J Environ Res Public Health. 2022 Aug 19;19(16):10310. doi: 10.3390/ijerph191610310 (PMC9408383; doi:10.3390/ijerph191610310)
Supplement: Supplementary file 1 [file ijerph-19-10310-s001.zip › ijerph-1844208-supplementary.pdf]

Table S1 Trauma Recovery Rubric

Directions: This rubric is designed to evaluate where a survivor is in their Trauma Recovery journey. These pathways represent the current state of the survivor on their trauma recovery journey overall and do not represent a permanent place of recovery. People may move in and out of these pathways depending on circumstances, but these are the dominant internal pathway the survivor on at the time of the interview.
While survivors may have had physical illnesses, other mental illnesses, had moved away from drugs, poverty, unemployment, homelessness, or other forms of hardship, this evaluation should focus on how much the survivor understands, wrestles with, reconciles, and integrates the impact of trauma in their life and health.

**Recovery pathway descriptions**	**Avoidance**<br>People in these pathways share a lack of awareness of how their trauma history relates to other aspects of their self, emotions, and functioning		**Disabling struggles to manage the effects of trauma**<br>These pathways share disabling responses as they engage in their trauma processing, but vary in the type of processing they engage in			**Regaining self-mastery and health**<br>These pathways share a degree of self and trauma understanding and mastery	
			**Magnification or internalization**<br>In this pathway, traumatic events dominate and define the survivor's life, and they focus on the disability they cause, often experiencing trauma as a damage to the self	**Overwhelmed**<br>People on these pathways share struggles to manage the effects of trauma, but they differ on how that struggle is approached, with some closing themselves off to feelings, and others shifting their focus outward to events in their environment.		**Integration**<br>People in these pathways have some self-understanding but differ in their ability to find peace and a sense of security within themselves. The seeker has hope that a state of peace is possible but is still experimenting and practicing. The integrated person is settled and secure because they know recovery will be a life's work; feeling equanimity which is a mental calmness, composure, and evenness of temper, especially in a difficult situation	
	**1-Normalizing**<br><br>Accepts social beliefs that normalize violence, and therefore does not feel experiences to be significant or important	**2-Minimizing**<br><br>Decreases the significance or does not recognize trauma and its aftermath on one's selfhood, functioning, emotions, and decisions by denying its significance	**3-Consumed/Trapped**<br><br>Views trauma and its aftermath as the primary determinant of one's selfhood, functioning, emotions, and decisions	**4- "Shut down" or frozen**<br><br>The "shut down" person seeks to close themselves off from feelings and their body<br><br>Cognitively recognizes trauma has an impact but unable to connect to trauma responses emotionally or physically; feels incapable of managing emotions or memories	**5-Surviving**<br><br>The "surviving" person focuses on events outside of themselves to explain their frustration and suffering<br><br>Feels out of control of their environment and their emotional responses to it; focuses on problems and solving them	**6-Seeking and fighting for integration**<br>Recovery efforts are primarily exploring ways to cope with or heal from the trauma; makes an intentional effort to find ways to heal	**7-Finding Integration/Equanimity**<br>Describes symptom mastery and successes in healing, but acknowledges and accepts *occasional* setbacks or negative emotions
**Domains**	**Recovery pathway domain criteria**						
**Trauma definition:** How the survivor defines the trauma she has experienced	○ Acknowledges that they experienced an unwanted or problematic event, but does not consider it traumatic and does not believe it was a big deal in the context of their life	○ Deny the trauma itself happened in their life	○ Focuses on trauma experiences to the exclusion of other life events. Finds it difficult to separate their selfhood from the trauma they have experienced; Reference their primary identity as a survivor of trauma.	○ Compartmentalizes or emotionally separates oneself from traumatic events; believes that they do not have the capacity to manage memories or feelings	○ Traumatic events are one of the numerous "bad things that have happened" that continually upset her; often describes themselves as attacked by trauma events, as well as everything that goes wrong; may think trauma events are the same as any other crisis event	○ Trying to put traumatic events into perspective, but feels it is an effort to do so; recognizes the importance of facing their trauma experience it is still an effort and difficult	○ Recognizes trauma and believes this is an important part of their recovery process; acknowledges that past traumatic events were abnormal and harmful, but attributes them to events and people outside of the self

**Balancing emotions, body, cognition, and behavior:** The extent to which the survivor can recognize and acknowledge the interactions among physical emotional mental and behavioral aspects of experiences	❍ May acknowledge physical or emotional pain to thinking or behavior, but believes it is irrelevant or insignificant; when they acknowledge that pain is related to GBV events, they believe they should not disclose or discuss it	❍ Thinks trauma does not affect their emotions and decision making and thus is unimportant. Does not admit to physical or emotional pain, or does not connect physical or emotional pain to thinking or behavior; Unable to connect emotions, needs, situations and interactions with others	❍ Vacillates between emotional and physical shutdown, or becoming overwhelmed by emotional and physical distress; emotionally labile; feels that the trauma is always centered in their perceptions of body, emotions, and cognitions; focus on how present body, emotions, and cognitions are dominated by trauma in the past	❍ Feels numb or disconnected to aspects of the self or emotions or their body; Acknowledges that trauma occurred, and may label event as problematic or troublesome, but emotionally separates from these events	❍ Chaos in daily emotional life feels unmanageable exhausting; does not relate physical or emotional pain to traumatic experiences specifically, because trauma is just one of the many things that have gone wrong	❍ Trying to detect patterns and experimenting with recovery strategies; it takes effort to contain or manage emotional or physical pain in order to be able to function; trying to learn to understand symptoms of body and emotions and the connection of those	❍ Consistently uses self-reflection to understand physical and emotional states; flexible about the connections between emotions, physical states, thinking and action
**Acceptance of trauma impact:** The degree of acceptance the survivor has for themselves as affected by trauma	❍ Does not acknowledge long term trauma effects in themselves or others, thinking that people should and can just get over it; Acknowledges some trauma effects but regards them as insignificant or unimportant	❍ Attributes GBV reactions to sources other than trauma	❍ Understands trauma impacts as a primary damage to selfhood; focuses on trauma effects as the primary driver in their emotions and relationships with others; struggles to separate current events from their trauma history; feels like they are now living in the past traumatic events	❍ Acknowledges some trauma effects exist within them, but regards them as burdensome; avoids self-evaluation of trauma impact to preserve functioning; avoids thinking about the impacts of the trauma to defend themselves or keep their functioning; believes self-evaluation of trauma impact is damaging; wants to keep the trauma in the past and move on without processing it	❍ Life events and survival are the focus of daily life; believes self-reflection about the impact of trauma is important or relevant to making life better; does not connect trauma to life chaos	❍ Trying to believe and hope that trauma impacts may eventually diminish someday but still finds it a struggle; trying to believe that their healing efforts now will go away someday	❍ Accepts trauma responses as a natural response to horrific events; recognizes it is natural and normal for emotions and feelings of security to be affected by trauma experiences
**Holistic self-view:** The degree that the survivor sees themselves as a whole person despite their struggles to recover from trauma	❍ Sees trauma as a personal flaw and minimizes its impact on everyday functioning	❍ Trivializes impact of trauma as irrelevant to their selfhood or the future	❍ Sees trauma events and trauma impact as the primary influence on themselves and their future; cannot see a future in which trauma impact will not be central	❍ Unable to feel or connect to the significance or trauma on themselves; notices themselves as unresponsive or emotionally disconnected or numb but unable to understand its source	❍ Chaos and emotional pain and frustration in their life is primarily attributed to life events; does not to recognize that they have capacity, skills, strengths, relationships, or resources; exhausted by the constant battle life presents	❍ Looking into the future is difficult, but making efforts to see themselves beyond the past; selfhood is fragile and can sometimes become dominated emotions or memories	❍ Consistently recognizes that they are more than their traumatic experiences; recognizes trauma effects as *part* of the self but not *all* the self; acknowledges both positive and negative parts of the self
**Autonomous Empowered functioning:** The degree of the survivor's	❍ Is unsure of their role in their event and thus also feels shame in discussing it; believes GBV is something that everyone	❍ Thinks it is their responsibility to avoid similar situations in the future	❍ Believes that trauma events and impacts are the central defining component of her functioning; cannot	❍ Unable to connect self, trauma, and functioning; believes functioning is the primary goal and keeps emotions and memories at	❍ Feels controlled by the chaos and emotional upset in their life; believes they are unable to control	❍ Hopeful that someday they can develop the capacity and skill to manage emotions and decisions; difficulty	❍ Believes that she has the responsibility and the capability take charge of her emotions and decisions

recognition that she has the capacity to manage her emotions and make her own decisions	faces and is just part of life		imagine a future in which her trauma does not impact her day-to-day interactions; believes her trauma responses impair her ability to function	bay to preserve functioning	life events or their responses to them	managing the healing needs with the needs to function	
**Engagement in a supportive social network:** The degree to which the survivor finds and engages authentically in supportive relationships	❍ Avoids discussing experiences because of shame and self-blame; May isolate socially to avoid discovery or risk of self-disclosure; avoids acknowledging needs related to shame and self-doubt	❍ Keeps their engagement with others superficial, or focused on situations or other's needs; keeps people away from their private lives	❍ Social relationships may be organized around survivorship and trauma recovery; may be unable to feel they can ever have satisfying relationships because of their trauma history; vacillates between needing help from others and withdrawal from others because symptoms are too severe or overwhelming; day-to-day interactions are generally shaped by trauma recovery experiences and needs	❍ Feels that they are somehow "separate" or "outside looking in" in relationships or in society; longs for connection but feels "cut off;" feels like a stranger, or an imposter; avoids close encounters to keep memories away	❍ Focuses on situations and chaos rather than relationships and closeness; wants others to solve their problems, or make it better; blames others for the crisis or chaos in their lives; unable to separate themselves from those who are difficult, cause conflict, or make them upset; mistrust and needs dominate their social relationships	❍ Focusing on boundaries in relationships; experimenting with ways to maintain boundaries but still be open to others; beginning to find others with whom they can feel secure and authentic; still struggling to trust themselves with others	❍ Evaluates need and vulnerability, and make appropriate judgements about interpersonal engagements; shares needs and feelings with trusted others; regularly engages in close and authentic relationships with trusted others; describes boundaries as a big part of how they keep themselves secure
**Number of criteria met**							
**Number assigned to a person with this dominant pathway**	**1**	**2**	**3**	**4**	**5**	**6**	**7**
**Holistic Reflection**	**In this section, write reflections about what you think, overall, affected this survivor's ability to integrate her trauma, or what you see as major obstacles or barriers to that integration. Only a short paragraph is necessary.**						
**Scoring:**	**There are 7 pathways. Assuming that none of the domains weigh more heavily, then we can use a simple range. Highest score =42; Lowest score =0**<br>**0-6 = may be in the normalizing pathway; 7-12 =may be in the minimizing pathway; 13-18 =may be in the consumed pathway; 19-24 = may be in the shutdown pathway; 25-30 = may be in the surviving pathway; 31-36 = may be in the fighting for integration pathway; 37-42 = may be in the integration pathway**						
